# Recalibrating Resting Energy Expenditure Prediction Equations in Asian Older Adults with Multimorbidity

**DOI:** 10.3390/nu18091345

**Published:** 2026-04-24

**Authors:** Pei San Kua, Musfirah Albakri, Su Mei Tay, Phoebe Si-En Thong, Olivia Jiawen Xia, Wendelynn Hui Ping Chua, Kevin Chong, Nicholas Wei Kiat Tan, Xin Hui Loh, Jia Hui Tan, Lian Leng Low

**Affiliations:** 1 SingHealth Community Hospitals, 10 Hospital Blvd, Singapore 168582, Singapore; musfirah.albakri@singhealthch.com.sg (M.A.); tay.su.mei@singhealthch.com.sg (S.M.T.); olivia.xia.j.w@singhealth.com.sg (O.J.X.); wendelynn.chua.h.p@singhealthch.com.sg (W.H.P.C.); kevin.chong@singhealthch.com.sg (K.C.); nicholas.tan.w.k@singhealthch.com.sg (N.W.K.T.); loh.xin.hui@singhealthch.com.sg (X.H.L.); low.lian.leng@singhealth.com.sg (L.L.L.); 2 SingHealth Centre for Population Health Research and Implementation, 10 Hospital Boulevard, #19-01 SingHealth Tower, Singapore 168582, Singapore

**Keywords:** prediction equations, resting energy expenditure, Asian older adults, multimorbidity, recalibration

## Abstract

Background/Objective: Accurate resting energy expenditure (REE) estimation is paramount for the nutritional management of older Asian adults with multimorbidity. However, standard predictive equations (PEs) lack precision for this cohort. This study aimed to recalibrate PEs using BMI-stratified, slope-only regression to enhance bedside accuracy. Methods: REE was measured via indirect calorimetry in 400 hospitalized patients (age ≥ 65). Sensitivity analyses identified significant proportional bias in existing models. Models were recalibrated and validated using 1000-iteration bootstrap resampling. Results: Standard PEs exhibited significant bias, particularly overpredicting requirements for 68% of underweight patients. The new Singapore Older Adults Resting energy expenditure (SOAR) PE 1 (963.67 + 8.56 × weight − 5.6 × age) eliminated weight-dependent systematic errors. The recalibrated models utilizing actual body weight achieved accuracy rates of up to 64% in obese cohorts, comparable to complex adjusted-weight protocols. Conclusions: Population-specific recalibration is essential to mitigate the bidirectional risks of malnutrition and overfeeding in geriatric rehabilitation. The BMI-stratified multipliers provided offer a robust, clinically efficient framework for individualized nutritional care.

## 1. Introduction

The global population of adults aged 65 and over has tripled since 1980 and is projected to more than 1.6 billion by 2050 [1]. Similarly, in Singapore, this demographic has grown from 11% in 2013 to 19% in 2023, with projections indicating that one in four adults will be 65 or older by 2030 [2]. With the increasing aging population and the effects of age-related body composition changes on nutritional health, assessment of accurate energy requirement is essential in clinical settings to improve nutritional management of older adults [3]. This allows for the maintenance or improvement of nutrition status, thus improving clinical outcomes in hospitalized older adults [4]. Measuring energy expenditure using indirect calorimetry (IC) remains a gold standard for dietary assessment [5]. However, IC is costly and not feasible in daily clinical practice [6]. As a result, clinicians frequently employ predictive equations as a time-efficient alternative for estimating REE in daily practice. These equations often require variables such as demographic data, anthropometric measurements, body composition parameters or specific clinical data [7].

While our previous research established several novel prediction equations (PEs) with superior accuracy for multi-ethnic Asian older adults with complex comorbidities [8], the present follow-up study aims to further refine these tools. We propose a recalibration procedure designed to enhance predictive precision across a cohort stratified by body mass index. We hypothesize that existing systematic inaccuracies are primarily driven by demographic discrepancies between the original derivation cohorts and our specific population, as well as BMI-dependent variations in REE [8,9].

## 2. Materials and Methods

The patient recruitment, data collection, and clinical measurement protocols for this cross-sectional study have been described in detail in our prior publication [8]. Briefly, this study recruited patients ≥ 65 years old who were receiving subacute and rehabilitation care at Outram Community Hospital (OCH) and Sengkang Community Hospital (SKCH) from March to December 2023. The participants were medically stable, able to eat orally, and could provide informed consent; exclusion criteria included age < 65, enteral feeding, and medical instability. This current analysis utilizes the same dataset to further validate commonly used REE predictive equations for hospitalized Asian older adults with multiple comorbidities. Data collected from patients included REE measurements that were collected using a Q-NRG^TM^ (COSMED Ltd., Rome, Italy), body weight, height, mid-upper arm circumference (MUAC) and calf circumference (CC). We also developed and assessed these equations to improve their predictive performance for our specific patient population. The study protocol received approval from the SingHealth Centralised Institutional Review Board (CIRB). All participants’ clinical characteristics were extracted from the Electronic Health Intelligence System (eHINTS) with prior ethics board approval.

### 2.1. Resting Energy Expenditure Using Predictive Equations

In addition to indirect calorimetry, REE was also estimated using a range of predictive equations (see Table 1). These equations were selected for their common use, development using older adults or Asian populations, or use of similar parameters such as age, weight, height, MUAC, CC and sex [8,10,11,12,13,14].

To evaluate our recalibrated models against standard clinical practices, a traditional weight-adjustment approach was implemented for the obese cohort (BMI ≥ 30 kg/m^2^). Under this method, the Mifflin–St Jeor [11] and Kawase [14] equations were calculated using actual body weight, as these PEs were originally derived from a cohort that included both non-obese and obese individuals. Conversely, the Harris–Benedict [10], Schofield [12] and weight-based [13] equations were calculated using adjusted body weight (ABW), defined as ABW = {Reference body weight} + 0.33 ({actual body weight} − {reference body weight}), where reference body weight represents the ideal body weight for the patient’s height [15].

### 2.2. Statistical Analysis

The sample size for this study was determined using the Cochran formula, (*n* = (Z_0.95_)^2^P[(1 − P)/D^2^]), a method appropriate for large populations, such as Singapore’s growing older adults’ demographic [16]. A prevalence of undernourishment of 39% among older patients in step-down and long-term care [17] was used as an estimate. This calculation yielded a minimum required sample size of 366 patients. Our final cohort of 400 participants surpassed this threshold, providing an adequate sample size for the study.

Accuracy of prediction was considered when REE predicted by the equations was within ± 10% of the measured REE [18]. Predictions were classified as underpredictions or overpredictions if the predicted REE deviated from the measured value by more than 10% in either direction [19]. The precision of each equation’s ability to predict REE, as measured by IC, was assessed using the coefficient of determination (R^2^) and the RMSE [20]. We evaluated both the original PEs and their recalibrated variants, which were adjusted through modifications to the intercept, the slope or both [21,22,23,24]. These recalibrations aimed to enhance predictive accuracy, as assessed by the RMSE, and reliability, as assessed by the interclass co-efficient (ICC) [25]. Adjustments were necessary because the original equations were developed from derivation cohorts that differ substantially from our study population [8,9,10,11,12,13,14]. Calibration plots were used to visually assess the agreement between predicted and measured REE, alongside the quantitative evaluation of RMSE and R^2^ values.

Normality of residuals was assessed using both the Shapiro–Wilk test and Q-Q plot inspection [26]. While the Shapiro–Wilk test provided a formal statistical assessment, its sensitivity to larger sample size (n = 397) warranted cautious interpretation [27]. Greater emphasis was placed on visual inspection of Q-Q plots, where approximate linearity of points along the diagonal line was taken as sufficient evidence of normality despite potentially significant test results [28]. Depending on the outcome of this assessment, either paired *t*-test (normally distributed data) or paired Wilcoxon signed-rank test (non-normally distributed data) was used to compare predicted REE values between the original and adjusted models [29]. To ensure model robustness and account for potential performance optimism, all equations underwent rigorous internal validation using bootstrap resampling with 1000 iterations. This process allowed for a stable estimation of predictive metrics in our specific cohort [30].

The analytical approach for this study was informed by our prior findings: although sex is traditionally included in REE PEs (Appendix A), our previous regression analysis on this cohort demonstrated that sex was not a statistically significant predictor of measured REE (*p* = 0.06), whereas body weight and height remained highly significant (*p* < 0.005) [10]. To evaluate the metabolic distinctness of the study population across various weight statuses, measured REE was compared based on the four BMI categories using a one-way analysis of variance (ANOVA) [31]. This analysis was essential to determine if significant physiological variations existed between the strata, thereby justifying the development of BMI-specific recalibration factors rather than a universal model [31]. In instances where the ANOVA revealed a significant overall effect, Tukey’s honestly significant difference (HSD) post hoc test was employed to identify specific pairwise differences between the BMI groups while controlling for Type I errors across multiple comparisons [32,33].

Furthermore, a sensitivity analysis was conducted to evaluate the robustness and clinical applicability of the baseline SOAR PE 1 and PE 2 across varying body compositions [34]. To quantify deviations, the mean bias was calculated for each BMI stratum [35]. Proportional bias was formally assessed via simple linear regression analysis, utilizing the residual error as the dependent variable and BMI as the independent variable [36]. A significance threshold of (*p* < 0.05) was applied to determine the presence of systematic proportional error. To evaluate the decay in predictive reliability at physiological extremes, the percentage of accurate predictions was isolated and compared within the underweight and obese cohorts. To address identified systematic errors and establish an accurate baseline formula prior to BMI-specific recalibrations, a multiple linear regression analysis was conducted on optimal steady-state data using weight and age as independent variables. Finally, agreement between measured and predicted REEs was evaluated using Bland–Altman analyses with limits of agreement (LOAs) for both the original and adjusted equations [37]. All statistical analyses were performed using R Studio (Version 2023.12.1 Build 402, RStudio Team, Boston, MA, USA) with a significance threshold maintained at *p* < 0.05.

## 3. Results

Based on our data shown in Table 2, it is observed that the median age is highest in the underweight group (82 years for males and 78 years for females) and progressively decreases across the normal, overweight, and obese categories. Regarding nutritional assessment, the median 7-point Subjective Global Assessment (SGA) scores are lower in the underweight group (5–6) and the maximum score of 7 is reached in the obese group. Physical measurements also show a clear progression across BMI categories; the median calf circumference is lowest in underweight individuals (27–29 cm) and highest in the obese category (39–40 cm). Similarly, it is observed that the median MUAC increased from 22–23 cm in the underweight group to 32–35 cm in the obese group. These trends are observed as the median BMI increases from 17 in the underweight group to 31–33 in the obese group.

Table 3 shows that specific disease distributions highlighted several disproportionalities when compared to the baseline population sizes. Although obese individuals comprised only 14.1% of the total cohort, they accounted for 33.3% of all reported gout cases. Conversely, while the underweight group made up 11.8% of the study population, this group accounted for 22.4% of all cancer and suspected cancer cases.

**Figure 1:**
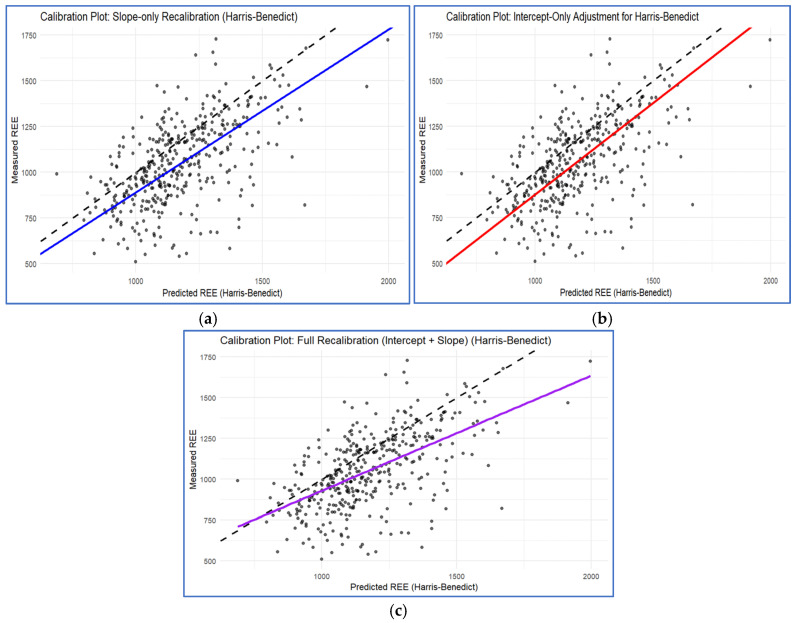
Calibration plots for Harris–Benedict [10] PE: comparison of slope-only (**a**), intercept-only (**b**), and combined slope–intercept (**c**) adjustments. The dashed line denotes perfect agreement (measured = predicted). Solid coloured lines show fitted calibration of the Harris–Benedict [10] equation (actual relationship between predicted and measured after adjustment).

Statistical analysis identified age, weight, height, 7-point SGA, MUAC, and calf circumference as significant predictors of measured REE (*p* < 0.05), as tabulated in Appendix A. We recalibrated the PEs by adjusting the slope, the intercept, or both; Figure 1 displays the calibration plots for the Harris–Benedict [10] equations, with remaining PEs are detailed in Appendix B. Notably, slope-only adjustments yielded improvements in predictive accuracy, as evidenced by improvements in R^2^ and RMSE, comparable to more complex recalibration methods. This approach offers a distinct clinical advantage: by utilizing a single multiplicative coefficient, the refined equations remain practical and memorable for bedside use. To ensure model robustness and account for performance optimism, all equations underwent internal validation using bootstrap resampling. Finally, the predictive performance of these adjusted models was validated against the original equations.

As weight was identified as a significant predictor in the derivation of the predictive equations, whereas sex did not demonstrate a significant association; therefore, subsequent stratified analyses were conducted based on weight rather than sex. Differences in REE were therefore examined across BMI categories. Prior to between-group comparisons, the distribution of measured REE was assessed and found to be approximately normal based on a visual inspection of the Q–Q plots. Homogeneity of variance across BMI categories was also confirmed. Consequently, differences in mean measured REE across BMI categories were evaluated using ANOVA. Where a significant overall effect was observed, Tukey’s post hoc test was applied to identify pairwise differences between BMI categories (Table 4).

The calibration of the eight REE PEs was executed using a slope-only linear regression model, and the results are tabulated in Table 5. This method yielded a singular calibration factor for each BMI subgroup, effectively adjusting the slope of the original equation to align with measured REE values. The data revealed significant systematic variances in equation performance based on weight status. The Schofield [12] and Kawase PE 1 and 2 [14] exhibited the most pronounced overestimation in underweight populations, requiring a downward calibration to 83% of their original values. Conversely, within the obese category, the weight-based PE [13] requires a 23% upward calibration, while the pre-calibrated SOAR PE 1 necessitates a 37% downward adjustment. In contrast, the SOAR PE 2 [8] equation demonstrated consistent accuracy across all BMI levels, maintaining a calibration factor near unity (1.00).

Sensitivity analysis revealed that baseline SOAR PE 1 exhibited significant proportional bias at BMI extremes (*p* < 0.05), with prediction errors increasing linearly as weight diverged from the median. Conversely, PE 2 avoided this weight-dependent error by utilizing alternative anthropometric parameters (e.g., circumferences) instead of body weight. While PE 1 performed acceptably for normal-weight adults, its stark inaccuracy in underweight adults statistically justified a drastic 1.65 recalibration multiplier. To resolve PE 1’s proportional bias, a multiple linear regression was first conducted on optimal steady-state data (using weight and age) to establish a more accurate baseline formula. Subsequently, BMI-specific recalibration multipliers were derived via slope-only linear regression, correcting the physiological weight–REE relationship without introducing an artificial intercept.

The resulting optimized model, new SOAR PE 1, can be expressed in standard linear form as 963.67 + 8.56 × weight − 5.6 × age. Application of these targeted multipliers effectively eliminated the weight-dependent systematic error across the full range of different body compositions. In the underweight cohort, implementation of the m = 0.99 multiplier increased predictive accuracy to 1%. Likewise, for the obese cohort (Table 5 and Table 6d), the m = 1.01 multiplier yielded the study’s peak predictive accuracy of 64%. By correcting the slope at physiological extremes, new SOAR PE 1 stabilizes predictive variance and yields a robust, clinically safe model that supports the use of actual body weight across all BMI categories.

The accuracy and predictive performance of the original and recalibrated REE PEs, stratified by BMI categories, are presented in Table 6a–d. Significant differences between the original and recalibrated equations were determined using the paired Wilcoxon signed-rank test (*p* < 0.05). In the underweight cohort (Table 6a), SOAR PE 2_R* emerged as the most accurate model, achieving a 47% accuracy rate with the lowest RMSE (160 kcal) and an ICC of 0.259. In contrast, the original Schofield [12] equation performed poorly, significantly overpredicting REE in 68% of cases with an accuracy of only 23%. For normal-weight participants (Table 6b), Kawase PEs 1 and 2_R* and new SOAR PE 1 provided reliable results with 44% accuracy. The original Schofield [12] equation was the least effective in this group, overpredicting REE in 63% of patients. In the overweight group (Table 6c), new SOAR PE 1_R* demonstrated the best performance with 52% accuracy and an ICC of 0.221. The original weight-based [13] equation remained the worst performer here, exhibiting a 65% underprediction rate and the lowest accuracy (27%).

To further evaluate the predictive performance of the PEs within the obese cohort, we compared traditional equations often calculated using adjusted body weight against our recalibrated formulas utilizing actual body weight. This approach aligns with the ESPEN practical guidelines for clinical nutrition and hydration in geriatrics, which recommend the use of actual body weight over adjusted body weight for older adults [15]. As demonstrated in Table 6d, the recalibrated models using actual body weight effectively mitigated systematic bias; for example, Harris–Benedict_R* showed a balanced error distribution (21% underprediction versus 21% overprediction). Furthermore, the recalibrated actual body weight model achieved a predictive performance (RMSE: 184; accuracy: 57%) comparable to that of the standard adjusted weight application (RMSE: 186; accuracy: 52%).

**Figure 2:**
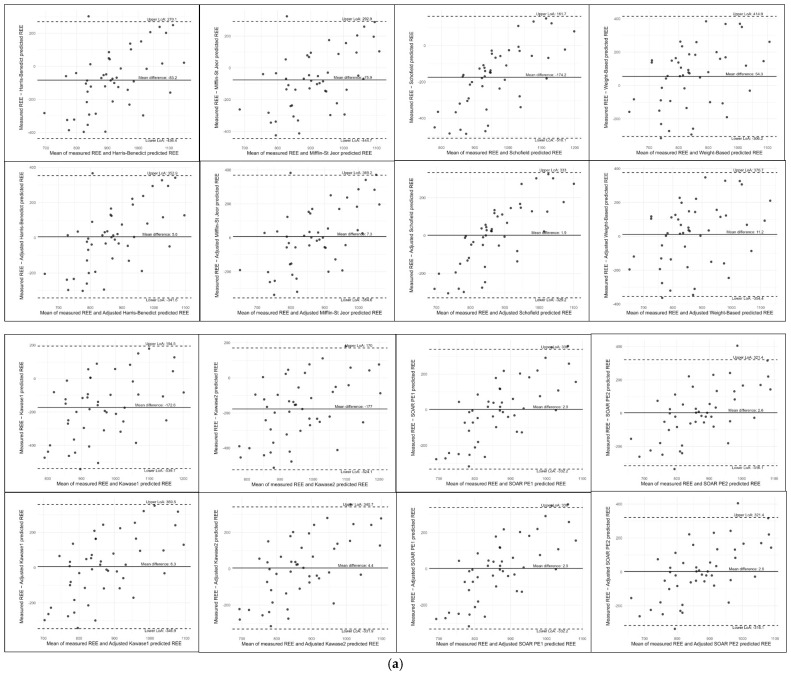

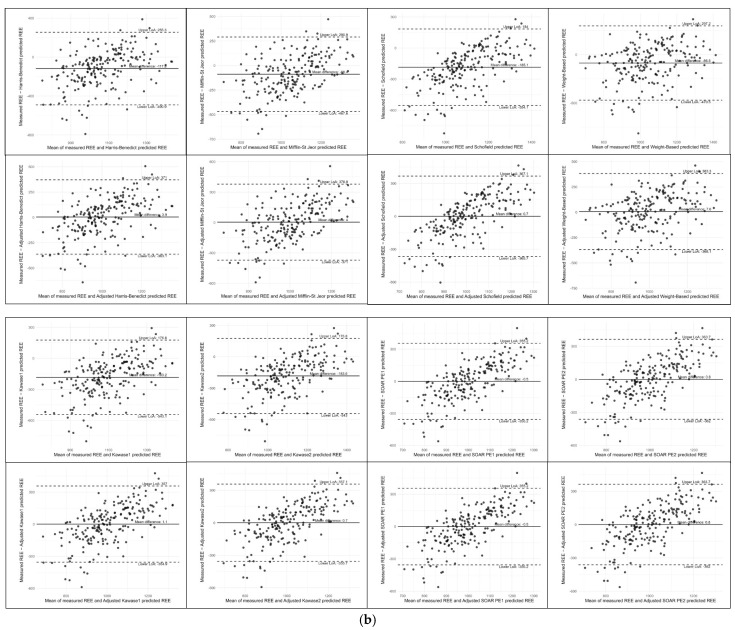

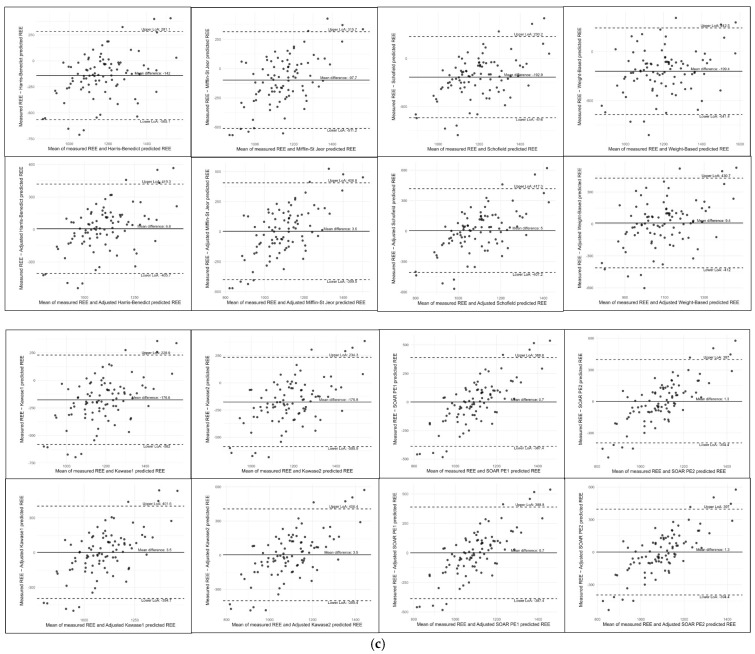

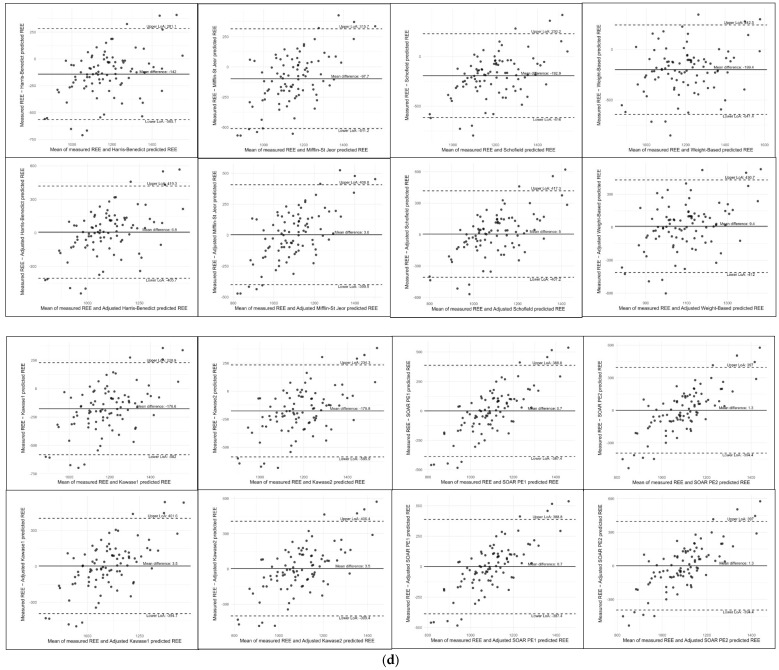
(**a**). Bland–Altman comparison of measured versus predicted REE in underweight patients using conventional and recalibrated equations. (**b**) Bland–Altman comparison of measured versus predicted REE in normal-weight patients using conventional and recalibrated equations. (**c**) Bland–Altman comparison of measured versus predicted REE in overweight patients using conventional and recalibrated equations. (**d**) Bland–Altman comparison of measured versus predicted REE in obese patients using conventional and recalibrated equations.

Bland–Altman analysis (Figure 2a–c) revealed that across all BMI groups, the recalibrated SOAR models were the most robust, maintaining a negligible mean bias (<3 kcal). Conversely, the Kawase equations [14] showed significant systematic underestimation in the underweight cohort (177 kcal). While recalibration successfully centered the mean for conventional models, it did not improve individual precision; all equations exhibited wide limits of agreement and proportional bias, consistently underestimating REE at lower metabolic rates and overestimating at higher rates.

Figure 2d revealed that while several standard models underestimated REE, most notably the weight-based equation [13] (mean bias: −367.8 kcal), the adjusted weight models (Rows 2 and 4) successfully mitigated systematic bias, bringing the mean values closer to zero. Despite this centering, precision remained poor across all equations, as evidenced by the wide limits of agreement (typically ±350–500 kcal) and a pervasive upward slope, which indicates a proportional bias where REE is underestimated at lower values and overestimated at higher values.

## 4. Discussion

### 4.1. Performance Comparison of Predictive Equations

To our knowledge, our present investigation represents the first study to recalibrate commonly utilized REE PEs specifically within a cohort of hospitalized Asian older adults with multimorbidity undergoing rehabilitation. Our findings highlight that these existing equations, which were primarily derived from younger or healthier populations, consistently lack the predictive precision required to accurately estimate the energy requirements of this specific patient demographic [8]. In alignment with prior literature, these inaccuracies are primarily characterized by a systematic overestimation across most BMI categories, and this phenomenon is largely explained by age-related physiological changes; as the fat-free mass, which has the most metabolically active tissues, declines with age, the REE relative to a given BMI typically decreases [38,39,40]. In particular, the original Schofield [12] and Kawase [14] equations demonstrated pronounced underprediction in the underweight cohort (BMI ≤ 18.5 kg/m^2^), failing to provide accurate energy requirements for 68% of our patients which is similar to the findings of previous studies [41,42]. Such discrepancies pose a significant clinical risk as overestimation can lead to overfeeding-related complications, while underestimation contributes to hospital malnutrition in an already vulnerable demographic [15,43,44].

In contrast, our newly developed Singapore Older Adults Resting energy expenditure (SOAR) PE 1 and previously published SOAR PE 2 demonstrated superior robustness across all weight statuses, even prior to recalibration [8]. These equations maintained higher internal validity and stability because they were developed specifically for the metabolic and body composition profiles of multi-ethnic Asian older adults with complex comorbidities [8]. Following adjustment, the SOAR models consistently outperformed traditional PEs, yielding the lowest RMSE and highest reliability (ICC) values, particularly in the obese category (BMI ≥ 30 kg/m^2^).

### 4.2. Importance of Recalibration

The importance of calibrating REE predictive equations lies in the need to bridge the gap between generalized models and the specific metabolic realities of clinical populations [10,15]. Without such recalibration, standard equations fail to provide the precise energy targets required to maintain muscle protein synthesis and attenuate muscle loss [45]. Our findings illustrate this precision gap where the original Schofield equation [12] underpredicted the requirements for 68% of underweight patients, creating a systematic risk of rehabilitation failure due to metabolic fuel deficits. This vulnerability is particularly critical given that these patients already exhibit significantly lower muscle mass, as evidenced by lower MUAC and CC measurements [46]. Conversely, the necessity of population-specific calibration is highlighted by the work of Kawase et al. [14], who showed that the same models tended to overestimate the needs of older Japanese inpatients. This bidirectional error of underestimation leading to poor recovery and overestimation, which leads to mobility-limiting weight gain underscores the fact that calibration is an essential intervention for navigating the complex nutritional demands of subacute and rehabilitation care for older adults [9].

While our SOAR prediction equations offer a robust foundation for multi-ethnic Asian older adults, applying generalized equations across varying body compositions can introduce complexity at the bedside. Furthermore, current ESPEN practical guidelines for clinical nutrition in geriatrics advocate for the use of actual body weight to streamline assessments and reflect true physiological load [15]. To eliminate the need for cumbersome ideal or adjusted body weight calculations, this study provides a new model, the new SOAR PE 1. As demonstrated in our sensitivity analysis, utilizing a static equation with actual body weight introduces severe proportional bias at BMI extremes. By applying the BMI-stratified multipliers developed herein directly to the patient’s actual weight, clinicians can achieve high predictive precision (64% accuracy in the obese cohort) through a simplified, single-step calculation that aligns with current best-practice guidelines.

### 4.3. Practicality of Slope-Only Recalibration

In our study, recalibration via a slope-only adjustment was prioritized over more complex intercept or combined methodologies. This preference stems from the physiological principle that modifying the slope corrects the fundamental relationship between physiological variables, whereas an intercept adjustment merely accounts for consistent and parallel bias [21,45]. From a clinical perspective, this approach facilitates direct bedside implementation, providing a single multiplicative coefficient that minimizes procedural complexity for healthcare professionals. These equations offer a clinically streamlined solution, remaining stable across all weight categories to allow for seamless integration into daily practice.

The absence of a linear trend in the percentage adjustments across BMI categories, as shown in Table 5, is a direct result of this slope-only methodology. Unlike an intercept-only approach, which would yield numerically consistent absolute energy shifts such as a fixed ± 100 kcal adjustment for all patients, slope recalibration specifically targets the rate of change in metabolic requirements relative to body mass [24]. While an intercept-only model may appear more uniform in a theoretical context, its primary clinical drawback is impracticality and practitioners would be burdened with memorizing and manually calculating distinct fixed-calorie additions or subtractions for every specific formula utilized [24].

To facilitate clinical implementation, healthcare professionals can utilize specific BMI categories to guide the adjustment of PEs, as shown in Table 5 and Table 7. For older patients within the underweight and normal-weight ranges (BMI ≤ 24.9 kg/m^2^), standard formulas frequently overestimate energy requirements, necessitating a reduction of approximately 10% to 15%, equivalent to applying a multiplier of between 0.85 and 0.90, which aligns with the metabolic findings in older Japanese populations [14]. In overweight patients, the Schofield [12] and weight-based [13] equations generally require a more modest downward adjustment of approximately 15% to 16%, which has been shown in a previous study [31].

While clinical practice has historically utilized adjusted body weight to mitigate overfeeding in obese populations, our findings demonstrate that a population-specific recalibration of the equation’s slope is both more accurate and clinically efficient [9,46]. This is underscored by recent evidence from the ESPEN practical guidelines, which state there is no clinical necessity to use adjusted body weight for older adults [16]. Using new SOAR PE 1 minimizes the complexity and potential for error that are inherent in multi-step ABW calculations. This evidence-based transition to actual weight simplifies the nutritional management of Asian older adults with multimorbidity while preventing the risk of both energy deficits and overfeeding.

For the obese category, the weight-based [13] equation tends to underestimate requirements and should be increased by approximately 23% (multiplier of 1.23), consistent with prior research in obese cohorts [9,47]. In contrast, the locally developed SOAR PE 1 and PE 2 models function as intrinsically stable frameworks; these equations maintain accuracy across the full BMI spectrum with minimal calibration requirements (~1%). Consequently, they facilitate direct bedside application by eliminating the need for computationally intensive auxiliary calculations, thereby streamlining nutritional assessment in daily clinical practice.

### 4.4. Strengths, Limitations and Future Research

Our work possesses several strengths that contribute to its clinical relevance. First, there were 400 participants, which surpassed the minimum required sample size of 366, ensuring that the study was sufficiently powered to evaluate predictive performance across diverse BMI categories [48]. Apart from that, the use of slope-only recalibration and internal validation via bootstrap resampling ensured the robustness of the predictive metrics while maintaining the ease of use necessary for bedside application [49]. Furthermore, the inclusion of the Kawase equation [14], which was specifically developed for older hospitalized patients in Japan, represents a significant strength of this study. By evaluating a model derived from a geographically and ethnically similar population of Asian older adults, this investigation provides a more contextually relevant comparison than studies relying exclusively on Western-derived formulas. This approach mirrors the methodology employed by Cai et al. [50], who similarly integrated Asian-specific models such as those by Liu et al. [51], Wang et al. [52] and Xue et al. [53] to enhance the cultural and physiological relevance of their finding.

However, several limitations must be acknowledged. The sample size for the underweight category (n = 47) was significantly smaller than the other groups, which may affect the generalizability and reliability of the performance metrics for this stratum [23,49]. In line with the recruitment barriers for vulnerable populations described by Sourial et al. [54], the enrollment of underweight patients in this investigation presented a significant challenge due to their inherent frailty and medical complexity [54,55]. Families frequently functioned as protective gatekeepers, expressing apprehension that the indirect calorimetry procedure despite being non-invasive might impose a fatiguing physical burden on their loved ones [56]. This heightened sensitivity to perceived participant burden contributed to disproportionately higher refusal and withdrawal rates, particularly among the most vulnerable and underweight individuals in our cohort [57]. While the recalibrated equations showed strong internal validity via bootstrap resampling, this study lacked external validation in independent Asian cohorts, which is necessary to confirm the broader clinical utility of the adjusted multipliers [48]. Additionally, as this study utilized a cross-sectional design focused on REE calibration, data on clinical outcomes such as length of hospital stay (LOS) were not captured. Future prospective studies are needed to evaluate how BMI-stratified recalibration influences hospital stay and overall cost-effectiveness in geriatric rehabilitation [58].

To enhance the clinical utility of these findings, future research should explore the integration of recalibrated SOAR PEs and adjusted standard models into Electronic Health Records (EHRs) and digital nutrition management platforms [58,59]. Aligning with current advancements in EHR-based machine learning and predictive assessment tools, such integration would allow for the automated application of population-specific multipliers based on a patient’s BMI [60]. This would significantly reduce the cognitive load on dietitians and clinicians, ensuring more consistent, evidence-based nutritional care at the bedside [57,60].

## 5. Conclusions

Our study establishes that locally developed SOAR equations offer superior stability and predictive validity across the full BMI spectrum, requiring minimal recalibration (≤3%) compared to traditional models. These results underscore the clinical imperative for population-specific recalibration via slope-only adjustments to mitigate the risk of energy deficits that impede rehabilitation or overfeeding that limits patient mobility. By utilizing the BMI-stratified multipliers validated herein, healthcare practitioners can achieve high predictive precision while utilizing actual body weight, thereby eliminating the computational burden and systematic errors associated with ideal weight calculations. Future research should prioritize external validation within independent Asian cohorts to confirm the cross-population utility of these adjusted multipliers and their long-term impact on geriatric clinical outcomes.

## Figures and Tables

**Table 1:** Established and novel resting energy expenditure (REE) predictive equations.

REE Predictive Equation (PE)	Formula (kcal/Day)	Description
Harris–Benedict [10]	M: 66.4730 + 13.7516 W + 5.0033 H − 6.7550 A F: 655.0955 + 1.8496 H + 9.5634 W − 4.6756 A	One of the earliest REE equations, developed using predominantly healthy Caucasian males (27 ± 9 years) and females (31 ± 14 years).
Mifflin–St Jeor [11]	9.99 W + 6.25 H − 4.92 A + 166 S (male = 1; female = 0) − 161	Developed using healthy, non-obese and obese American adults (mean age 45 ± 14 years).
Schofield [12]	M: 11.711 W + 587.7 F: 9.082 W + 658.5	Developed using European and North American populations with a large proportion of Italian subjects (47%), ranging from infants to adults > 60 years.
Weight-based [13]	18–20 kcal/kg	Developed using older hospitalized patients in acute medical wards aged > 65 years with at least 2 co-occurring chronic diseases present in at least 50% of the study population or the mean number of diseases or drugs/medication or the Charlson comorbidity index in the study population was more than 1.5.
Kawase [14]	PE 1: 313.582 + 3.973 H + 5.332 W − 5.474 A − 20.012 S (0 = male; 1 = female) + 12.174 CC PE 2: 3.760 H + 8.888 W − 6.298 A − 16.396 S (male = 0; female = 1) + 594.819	Developed using older hospitalized Japanese patients aged 70–102 years.
Singapore Older Adults Resting Energy Expenditure PE 1 [8]	2420.11 + 34.13 W − 5.65 A − 18.31 H	Developed using older hospitalized Singaporean patients with multimorbidity aged 65–100 years.
Singapore Older Adults Resting Energy Expenditure PE 2 [8]	812.67 + 12.61 M + 13.65 CC − 57.5 S (male = 0; female = 1) − 6.99 A	

W = body weight (kg), H = height (cm), A = age (years), M = mid-upper arm circumference (cm), CC = calf circumference (cm); obese = body mass index ≥ 30kg/m^2^, S = sex where male = 0; female = 1, PE = predictive equation.

**Table 2:** Patient characteristics.

	Underweight (≤18.4 kg/m^2^), n = 47				Normal (18.5–24.9 kg/m^2^), n = 203				Overweight (25.0–29.9 kg/m^2^), n = 91				Obese (≥30 kg/m^2^), n = 56			
	Male		Female		Male		Female		Male		Female		Male		Female	
	Med ^#^	IQR *	Med ^#^	IQR *	Med ^#^	IQR *	Med ^#^	IQR *	Med ^#^	IQR *	Med ^#^	IQR *	Med ^#^	IQR *	Med ^#^	IQR *
Age (year)	82	70–84	78	70–85	75	70–82	74	69–79	74	73–79	73	68–78	71	69–75	70	68–75
Weight (kg)	45	42–50	39	36–41	58	53–64	52	48–55	73	69–75	60	57–65	85	80–87	79	74–86
Height (cm)	167	162–169	152	148–155	165	160–170	154	149–157	165	160–167	151	147–153	164	160–168	153	148–155
7-point Subjective Global Assessment (SGA)	5	4–6	6	5–6	6	5–6	6	6–7	7	6–7	6	6–7	7	6–7	7	6–7
Calf circumference (CC, cm)	29	26–30	27	25–28	32	30–34	31	29–32	36	34–37	34	32–35	39	37–41	40	37–42
Mid-Upper Arm Circumference (MUAC, cm)	23	22–24	22	21–23	27	25–29	26	25–28	30	28–32	30	28–32	32	32–35	35	32–37

Notes: * IQR = Interquartile Range, ^#^ Med = median.

**Table 3:** Prevalence of common comorbidities across BMI categories in the study population.

Common Comorbidity	Total n = 397	Underweight n = 47	Normal Weight n = 203	Overweight n = 91	Obese n = 56
Hypertension	289 (72.8%)	26 (9%)	145 (50.2%)	74 (25.6%)	44 (15.2%)
Hyperlipidemia	247 (62.2%)	21 (8.5%)	127 (51.4%)	66 (26.7%)	33 (13.4%)
Diabetes Mellitus	143 (36%)	10 (7%)	69 (48.3%)	42 (29.4%)	22 (15.4%)
Gout	33 (8.3%)	2 (6.1%)	12 (36.3%)	8 (24.2%)	11 (33.3%)
Kidney Disease	105 (26.4%)	12 (11.4%)	52 (49.5%)	25 (23.8%)	16 (15.2%)
Cancer and Suspected Cancer	67 (16.9%)	15 (22.4%)	34 (50.7%)	12 (17.9%)	6 (9%)
Cardiovascular Diseases	154 (38.8%)	17 (11%)	78 (50.6%)	39 (25.3%)	20 (13%)
Cerebrovascular Diseases	135 (34%)	16 (11.9%)	71 (52.6%)	29 (21.5%)	19 (14.1%)
Liver Disease	35 (8.8%)	5 (14.3%)	19 (54.3%)	4 (11.4%)	7 (20%)
Bone Surgery or Fractures	250 (63%)	24 (9.6%)	121 (48.4%)	65 (26%)	40 (16%)
Osteoporosis and Osteoarthritis	214 (53.9%)	21 (9.8%)	98 (45.8%)	60 (28%)	35 (16.4%)
Respiratory Disease	97 (24.4%)	11 (11.3%)	50 (51.5%)	20 (20.6%)	16 (16.5%)
Number of Comorbidities (mean, IQR)	5 (4–7)	4 (3–6)	5 (4–7)	6 (5–7)	6 (5–7)

**Table 4:** Tukey’s post hoc test for the comparison of estimated energy requirements for different BMI categories.

BMI		BMI	MD (kcal/Day)	95% CI (Lower)	95% CI (Upper)	*p*-Value (Tukey)
Underweight	-	Normal weight	−130.5	−213.9	−47.1	<0.001
	-	Overweight	−225.8	−318.4	−133.2	<0.001
	-	Obese	−410.4	−512.3	−308.4	<0.001
Normal weight	-	Overweight	−95.3	−160.3	−30.3	<0.001
	-	Obese	−279.9	−357.7	−202.1	<0.001
Overweight	-	Obese	−184.6	−272.1	−97.1	<0.001

Notes: MD = Mean Difference; CI = confidence interval.

**Table 5:** Calibration factors and percentage adjustments.

Recalibrated PE	Underweight	Normal Weight	Overweight	Obese
Harris–Benedict_R*	0.90723 × PE (−9%)	0.89154 × PE (−11%)	0.88020 × PE (−12%)	0.89672 × PE (−10%)
Schofield_R*	0.83198 × PE (−17%)	0.84387 × PE (−16%)	0.84692 × PE (−15%)	0.88644 × PE (−11%)
Mifflin–St Jeor_R*	0.91238 × PE (−9%)	0.91553 × PE (−8%)	0.91478 × PE (−8 9%)	0.91247 × PE (−9%)
Weight-based_R*	1.05268 × PE (5%)	0.91366 × PE (−9%)	0.83930 × PE (−16%)	0.77244 × PE (−23%)
Kawase1_R*	0.82906 × PE (−17%)	0.84486 × PE (−16%)	0.85896 × PE (−14%)	0.88795 × PE (−11%)
Kawase2_R*	0.82742 × PE (−17%)	0.84490 × PE (−16%)	0.85943 × PE (−14%)	0.89412 × PE (−11%)
SOAR PE 1 (not recalibrated)	1.65016 × PE (65%)	1.01772 × PE (2%)	0.79702 × PE (−20%)	0.63359 × PE (−37%)
New SOAR PE 1_R*	0.99233 × PE (no adjustment needed)	0.99711 × PE (no adjustment needed)	0.99696 × PE (no adjustment needed)	1.0135 × PE (1%)
SOAR PE 2_R*	0.98660 × PE (−1%)	0.98869 × PE (−1%)	0.99803 × PE (no adjustment needed)	1.03570 × PE (3%)

Note: R* = recalibrated; PE = predictive equation. The percentages in parentheses indicate the total adjustment (increase or decrease) applied to the results of the original formulas.

**Table 6:** (**a**) performance of PEs in patients with a BMI ≤ 18.5 kg/m^2^ (n = 47). (**b**) performance of PEs in patients with a BMI between 18.6 and 24.9 kg/m^2^ (n = 203). (**c**) performance of PEs in patients with a BMI between 25 and 29.9 kg/m^2^ (n = 91). (**d**) performance of PEs in patients with a BMI ≥ 30.0 kg/m^2^ (n = 56).

(**a**)							
**Prediction Equation**	**REE (kcal)**	**Accurate** **Prediction (%) ^i^**	**Underprediction (%) ^ii^**	**Overprediction (%) ^iii^**	**R^2^**	**RMSE (kcal) ^iv^**	**ICC [95% CI] ^v^**
IC	874.1 ± 169.2						
Harris–Benedict [10]	957.2 ± 93.9	38%	15%	47%	−0.43	196	0.114 ⬤ [−0.129, 0.36]
Harris–Benedict_R*	868.4 ± 85.2	43%	30%	27%	−0.15	175	0.128 ⬤ [−0.169, 0.401]
Mifflin–St Jeor [11]	950.0 ± 95.0	36%	17%	47%	−0.51	200	0.0528 ⬤ [−0.193, 0.309]
Mifflin–St Jeor_R*	866.8 ± 86.7	43%	30%	27%	−0.24	182	0.058 ⬤ [−0.237, 0.34]
Schofield [12]	1048.3 ± 71.3	23%	9%	68%	−1.20	243	0.0684 ⬤ [−0.09, 0.261]
Schofield_R*	872.2 ± 59.3	40%	32%	28%	0.03	166	0.114 ⬤ [−0.182, 0.39]
Weight-based [13]	819.7 ± 110.4	23%	47%	30%	−0.34	188	0.162 ⬤ [−0.109, 0.418]
Weight-based_R*	862.9 ± 116.2	32%	38%	30%	−0.27	184	0.177 ⬤ [−0.118, 0.441]
Kawase PE 1 [14]	1046.7± 103.2	26%	6%	68%	−1.41	252	0.0615 ⬤ [−0.101, 0.258]
Kawase PE 1_R*	867.8 ± 85.6	40%	28%	32%	−0.17	177	0.0987 ⬤ [−0.198, 0.376]
Kawase PE 2 [14]	1051.1 ± 100.7	28%	4%	68%	−1.32	248	0.107 ⬤ [−0.084, 0.322]
Kawase PE 2_R*	869.7 ± 83.3	40%	32%	28%	−0.06	169	0.175 ⬤ [−0.121, 0.441]
New SOAR PE 1	878.0 ± 67.5	43%	26%	31%	−0.05	169	0.12 ⬤ [−0.176, 0.395]
New SOAR PE 1_R*	871.2 ± 67.0	45%	28%	27%	−0.05	169	0.12 ⬤ [−0.177, 0.394]
SOAR PE 2 [8]	883.2 ± 84.1	45%	28%	27%	0.05	161	0.261 ◆ [−0.029, 0.509]
SOAR PE 2_R*	871.4 ± 83.0	47%	28%	25%	0.05	160	0.259 ◆ [−0.032, 0.509]
(**b**)							
IC	1004.5. ± 202.4						
Harris–Benedict [10]	1122.3 ± 132.0	40%	9%	51%	−0.23	223	0.307 ◆ [0.095, 0.477]
Harris–Benedict_R*	1000.6 ± 117.7	44%	28%	28%	0.14	186	0.361 ◆ [0.235, 0.475]
Mifflin–St Jeor [11]	1092.9 ± 121.3	40%	13%	47%	−0.11	212	0.288 ◆ [0.131, 0.426]
Mifflin–St Jeor_R*	1000.6 ± 111.0	41%	29%	30%	0.10	191	0.314 ◆ [0.184, 0.433]
Schofield [12]	1189.6 ± 101.9	35%	2%	63%	−0.72	263	0.186 ⬤ [−0.042, 0.387]
Schofield_R*	1003.9 ± 86.0	40%	29%	31%	0.14	186	0.278 ◆ [0.146, 0.401]
Weight-based [13]	1091.2 ± 152.7	45%	13%	42%	−0.14	214	0.362 ◆ [0.2, 0.498]
Weight-based_R*	997.0 ± 139.5	42%	32%	26%	0.09	191	0.393 ◆ [0.27, 0.503]
Kawase PE 1 [14]	1187.7 ± 119.3	34%	2%	64%	−0.67	259	0.243 ◆ [−0.044, 0.457]
Kawase PE 1_R*	1003.5 ± 100.8	44%	28%	28%	0.19	181	0.343 ◆ [0.216, 0.459]
Kawase PE 2 [14]	1188.1 ± 114.0	35%	2%	63%	−0.66	259	0.232 ◆ [−0.044, 0.468]
Kawase PE 2_R*	1003.9 ± 96.3	44%	28%	29%	0.19	181	0.351 ◆ [0.225, 0.467]
New SOAR PE 1	1007.9 ± 84.4	44%	26%	30%	0.19	181	0.316 ◆ [0.187, 0.435]
New SOAR PE 1_R*	1005.0 ± 84.2	43%	27%	30%	0.19	181	0.316 ◆ [0.186, 0.435]
SOAR PE 2 [8]	1015.2 ± 92.6	44%	24%	32%	0.16	185	0.308 ◆ [0.179, 0.428]
SOAR PE 2_R*	1003.7 ± 91.6	42%	27%	31%	0.16	185	0.307 ◆ [0.177, 0.427]
(**c**)							
IC	1099.0 ± 209.6						
Harris–Benedict [10]	1241.9 ± 137.6	41%	8%	52%	−0.58	256	0.197 ⬤ [−0.008, 0.388]
Harris–Benedict_R*	1093.1 ± 121.1	51%	25%	24%	−0.02	207	0.246 ◆ [0.042, 0.43]
Mifflin–St Jeor [11]	1197.6 ± 109.7	42%	12%	46%	−0.28	231	0.177 ⬤ [−0.011, 0.357]
Mifflin–St Jeor_R*	1095.5 ± 100.4	44%	30%	26%	−0.007	206	0.196 ◆ [−0.011, 0.387]
Schofield [12]	1292.7 ± 122.9	29%	4%	67%	−0.98	290	0.13 ⬤ [−0.053, 0.314]
Schofield_R*	1094.8 ± 104.1	45%	27%	27%	−0.03	208	0.194 ⬤ [−0.013, 0.385]
Weight-based [13]	1299.3 ± 161.5	27%	8%	65%	−1.15	299	0.176 ⬤ [−0.044, 0.381]
Weight-based_R*	1090.5 ± 135.6	51%	24%	25%	−0.08	214	0.26 ◆ [0.057, 0.442]
Kawase PE 1 [14]	1276.5 ± 118.4	32%	8%	62%	−0.72	270	0.171 ⬤ [−0.041, 0.372]
Kawase PE 1_R*	1096.4 ± 101.7	51%	24%	25%	0.04	202	0.242 ◆ [0.037, 0.427]
Kawase PE 2 [14]	1275.7 ± 113.1	33%	5%	62%	−0.74	271	0.149 ⬤ [−0.046, 0.339]
Kawase PE 2_R*	1096.4 ± 97.2	49%	26%	25%	0.03	203	0.21 ◆ [0.004, 0.399]
New SOAR PE 1	1102.5 ± 79.6	52%	21%	27%	0.09	195	0.222 ◆ [0.016, 0.409]
New SOAR PE 1_R*	1099.2 ± 79.3	52%	21%	27%	0.09	196	0.221 ◆ [0.015, 0.409]
SOAR PE 2 [8]	1100.7 ± 78.2	47%	26%	26%	0.06	200	0.187 ⬤ [−0.02, 0.379]
SOAR PE 2_R*	1098.6 ± 78.0	47%	27%	25%	0.06	200	0.187 ⬤ [−0.021, 0.379]
(**d**)							
REE	1284.4 ± 196.6						
True/Actual Weight							
Harris–Benedict [10]	1424.6 ± 165.6	52%	2%	46%	−0.51	234	0.339 ◆ [0.028, 0.578]
Harris–Benedict_R*	1277.4 ± 148.5	57%	21%	21%	0.08	184	0.43 ◆ [0.188, 0.622]
Mifflin–St Jeor [11]	1400.1 ± 145.7	46%	7%	46%	−0.44	230	0.261 ◆ [0.01, 0.484]
Mifflin–St Jeor_R*	1277.5 ± 132.9	54%	25%	21%	−0.03	193	0.31 ◆ [0.051, 0.53]
Schofield [12]	1442.9 ± 138.2	48%	2%	50%	−0.74	251	0.218 ◆ [−0.04, 0.455]
Schofield_R*	1279.0 ± 122.5	54%	23%	23%	0.01	190	0.3 ◆ [0.039, 0.522]
Weight-based [13]	1642.3 ± 222.7	14%	0%	86%	−4.26	436	0.109 ⬤ [−0.076, 0.324]
Weight-based_R*	1268.6 ± 172.0	48%	30%	21%	−0.32	219	0.269 ◆ [0.006, 0.496]
Kawase PE 1 [14]	1443.5 ± 129.6	41%	2%	57%	−0.61	243	0.272 ◆ [−0.035, 0.525]
Kawase1_R*	1281.7 ± 115.1	62%	18%	20%	0.14	176	0.377 ◆ [0.126, 0.582]
Kawase PE 2 [14]	1435.3 ± 110.4	48%	2%	50%	−0.48	232	0.253 ◆ [−0.035, 0.5]
Kawase PE 2_R*	1283.3 ± 98.7	61%	18%	21%	0.16	175	0.345 ◆ [0.089, 0.557]
New SOAR PE 1	1264.6 ± 106.0	64%	20%	16%	0.09	183	0.316 ◆ [0.06, 0.533]
New SOAR PE 1_R*	1281.7 ± 107.4	61%	20%	20%	0.20	181	0.321 ◆ [0.062, 0.538]
SOAR PE 2 [8]	1237.1 ± 96.9	55%	31%	14%	−0.02	193	0.231 ◆ [−0.021, 0.459]
SOAR PE 2_R*	1281.3 ± 100.4	54%	23%	23%	0.03	189	0.247 ◆ [−0.018, 0.479]
Adjusted Weight							
Harris–Benedict [10]	1204.4 ± 109.9	52%	38%	11%	0.08	186	0.386 ◆ [0.131, 0.591]
Schofield [12]	1240.4 ± 90.3	50%	36%	14%	0.07	188	0.265 ◆ [0.013, 0.487]
Weight-based [13]	1224.8 ± 125.1	50%	36%	14%	−0.028	198	0.318 ◆ [0.073, 0.529]
New SOAR PE 1 [8]	1085.9 ± 64.1	20%	75%	5%	−0.798	261	0.164 ⬤ [−0.079, 0.409]

Notes for Table 6a–d: R* = recalibrated. ^i^ The percentage of participants predicted by this predictive equation within 10% of the measured value. ^ii^ The percentage of participants predicted by this predictive equation within <10% of the measured value. ^iii^ The percentage of participants predicted by this predictive equation within >10% of the measured value. ^iv^ Root mean squared prediction error (RMSE). ^v^ Intraclass correlation coefficient (ICC, with 95% confidence interval): ◆ 0.21–0.40: fair agreement; ⬤ ≥ 0.2: poor or slight agreement.

**Table 7:** Recommended predictive equations for resting energy expenditure (REE) across body mass index (BMI) categories.

BMI Category	Suggested Formula	Clinical Rationale
Underweight (≤18.4 kg/m^2^)	New SOAR PE 1: 963.67 + 8.56 W − 5.6 A	Highest baseline accuracy; minimizes underprediction
Normal Weight (18.5–24.9 kg/m^2^)	New SOAR PE 1: 963.67 + 8.56 W − 5.6 A	Lowest RMSE; corrects systematic overprediction
Overweight (25.0–29.9 kg/m^2^)	SOAR PE 2: 812.67 + 12.61 M + 13.65 CC − 57.5 S − 6.99 A	Optimized for moderate adiposity variance
Obese (≥30.0 kg/m^2^)	New SOAR PE 1: 963.67 + 8.56 W − 5.6 A	Least adjustment required; strongest alignment with ESPEN guidelines [15]

W = actual body weight (kg), A = age (years), M = mid-upper arm circumference (cm) and CC = calf circumference (cm) and S = sex where male = 0; female = 1.

**Figure A1:**
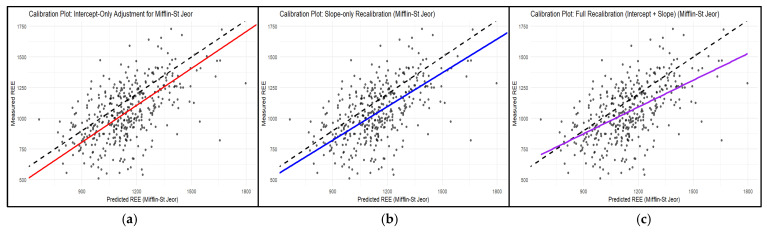
Calibration plots for Mifflin St–Jeor [11] PE: comparison of intercept-only (**a**), slope-only (**b**), and combined slope–intercept (**c**) adjustments. The dashed line denotes perfect agreement (measured = predicted). Solid coloured lines show fitted calibration of the Mifflin St–Jeor [11] equation (actual relationship between predicted and measured after adjustment).

**Figure A2:**
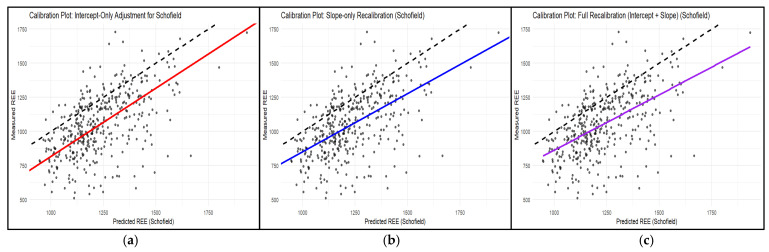
Calibration plots for Schofield [12] PE: comparison of intercept-only (**a**), slope-only (**b**), and combined slope–intercept (**c**) adjustments. The dashed line denotes perfect agreement (measured = predicted). Solid coloured lines show fitted calibration of the Schofield [12] equation (actual relationship between predicted and measured after adjustment).

**Figure A3:**
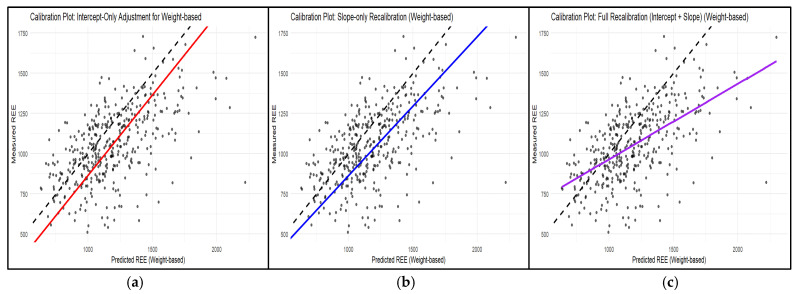
Calibration plots for weight-based [13] PE: comparison of intercept-only (**a**), slope-only (**b**), and combined slope–intercept (**c**) adjustments. The dashed line denotes perfect agreement (measured = predicted). Solid coloured lines show fitted calibration of the weight-based [13] equation (actual relationship between predicted and measured after adjustment).

**Figure A4:**
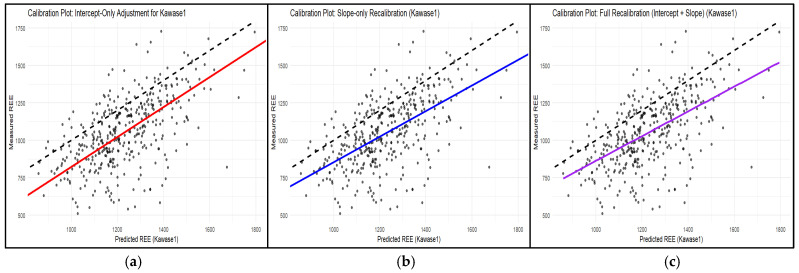
Calibration plots for Kawase PE 1 [14]: comparison of intercept-only (**a**), slope-only (**b**), and combined slope–intercept (**c**) adjustments. The dashed line denotes perfect agreement (measured = predicted). Solid coloured lines show fitted calibration of the of Kawase PE 1 [14] equation (actual relationship between predicted and measured after adjustment).

**Figure A5:**
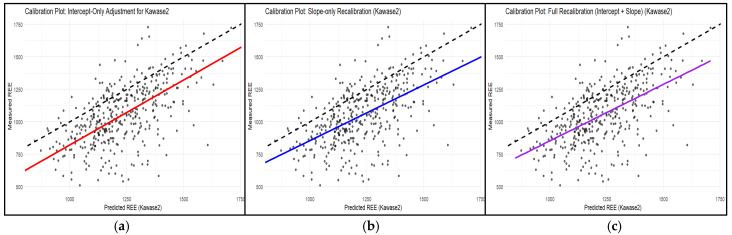
Calibration plots for Kawase PE 2 [14]: comparison of intercept-only (**a**), slope-only (**b**), and combined slope–intercept (**c**) adjustments. The dashed line denotes perfect agreement (measured = predicted). Solid coloured lines show fitted calibration of the of Kawase PE 2 [14] equation (actual relationship between predicted and measured after adjustment).

**Figure A6:**
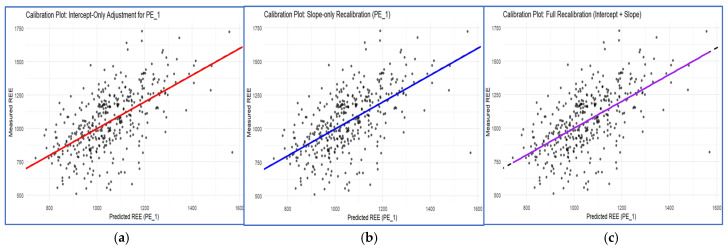
Calibration plots for new SOAR PE 1: comparison of intercept-only (**a**), slope-only (**b**), and combined slope–intercept (**c**) adjustments. The dashed line denotes perfect agreement (measured = predicted). Solid coloured lines show fitted calibration of the new SOAR PE 1 equation (actual relationship between predicted and measured after adjustment).

**Figure A7:**
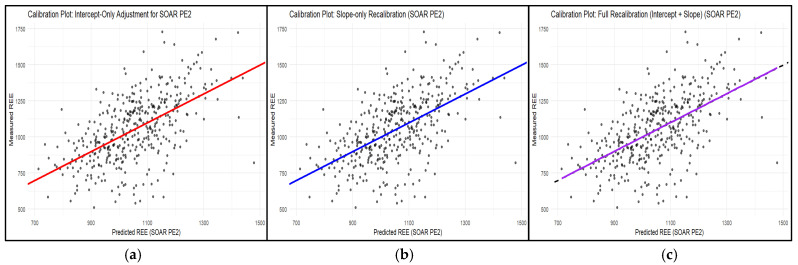
Calibration plots for SOAR [8] PE 2: comparison of intercept-only (**a**), slope-only (**b**), and combined slope–intercept (**c**) adjustments. The dashed line denotes perfect agreement (measured = predicted). Solid coloured lines show fitted calibration of the SOAR [8] PE 2 equation (actual relationship between predicted and measured after adjustment).

## Data Availability

The raw data supporting the conclusions of this article will be made available by the authors on request.

## Abbreviations

The following abbreviations are used in this manuscript:

REE	Resting energy expenditure
PE	Prediction equation
MUAC	Mid-upper arm circumference
CC	Calf circumference
SGA	Subjective Global Assessment
BMI	Body mass index

## Appendix A

**Table A1:** Regression analysis of factors associated with energy requirements.

Variable	Estimate (95% Confidence Interval)	*p*-Value
Age	−11.05 (−14/22, −7.89)	<0.005
Weight	14.60 (11.59, 17.60)	<0.005
Height	5.11 (2.60, 7.61)	<0.005
7-point SGA	93.81 (67.48, 120.15)	<0.005
Mid-Upper Arm Circumference	24.82 (20.51, 29.12)	<0.005
Calf Circumference	26.89 (22.26, 31.51)	<0.005
Sex (Female)	−44.15 (−90.59, 2.28)	0.06
